# Subtotal colectomy with antiperistaltic caecorectal anastomosis versus total colectomy with ileorectal anastomosis for refractory slow-transit constipation: a comparative analysis

**DOI:** 10.1007/s13304-026-02788-3

**Published:** 2026-08-24

**Authors:** Ugo Grossi, Alice Pecorino, Sayali Valiyeva, Nicola Passuello, Carla Felice, Matteo Fassan, Emanuela Tessari, Luca Faccio, Antonio Rella, Renato Pietroletti, Giacomo Sarzo

**Affiliations:** 1 Proctology and Pelviperineology Unit, Humanitas Gavazzeni and Castelli, Via Mauro Gavazzeni 21, 24125 Bergamo, Italy; 2 OSA General Surgery, Padua University Hospital, Padua, Italy; 3 Department of Applied Clinical and Biotechnological Sciences, Surgical Coloproctology, University of L’Aquila, Hospital Val Vibrata, Sant’Omero, Teramo, Italy; 4 Internal Medicine Unit, Regional Hospital Treviso, Piazzale dell’Ospedale 1, 31100 Treviso, Italy; 5 Department of Medicine (DIMED), University of Padova, Padua, Italy; 6 Veneto Institute of Oncology, IOV-IRCCS, Padua, Italy

**Keywords:** Slow-transit constipation, Colectomy, Anastomosis, Caecorectal, Functional outcomes

## Abstract

In selected cases of slow-transit constipation (STC), total colectomy with ileorectal anastomosis (TC-IRA) remains the standard surgical treatment but is associated with heterogeneous outcomes and relevant functional sequelae. Subtotal colectomy with caecorectal anastomosis (SCCRA) has been proposed as an alternative approach aiming to preserve the ileocaecal junction and improve postoperative bowel function. A prospective bicentre cohort of patients undergoing minimally invasive SCCRA for refractory STC was analysed and compared with a retrospective cohort of patients undergoing TC-IRA. Primary outcome was change in PAC-QOL score at 6 months. Secondary outcomes included PAC-SYM, St Mark’s incontinence score, bowel movement frequency, and postoperative outcomes. Histopathological findings from colectomy specimens in the SCCRA cohort were also evaluated. Eleven patients underwent SCCRA and 21 underwent TC-IRA. In the SCCRA cohort, PAC-QOL and PAC-SYM scores significantly improved at 6 months (*p* < 0.001 and *p* = 0.004, respectively), with bowel movements increasing from a median of 1 to 7 per week (*p* = 0.003), without deterioration in continence. One anastomotic leak required reoperation. Compared with SCCRA, TC-IRA patients had higher baseline disease burden (PAC-QOL 3.88 vs. 2.00, *p* < 0.001) and greater absolute improvement in PAC-QOL (Δ3.28 vs. 1.54, *p* < 0.001). However, TC-IRA was associated with significantly higher postoperative bowel frequency (21 vs. 7/week, *p* = 0.002). Operative time and baseline characteristics were comparable. Histopathological evaluation of the SCCRA cohort revealed a heterogeneous spectrum of pathological alterations. Minimally invasive SCCRA provides significant functional improvement while maintaining a more physiological bowel frequency. However, direct comparison with TC-IRA is limited by differences in baseline disease burden and study design, and these findings should be confirmed in prospective comparative studies.

## Introduction

Chronic constipation (CC) is a common gastrointestinal disorder, with an estimated prevalence of approximately 10–15% in the general population, and represents a significant cause of morbidity, impaired quality of life, and healthcare utilization [1]. Among the different CC phenotypes, slow-transit constipation (STC) is the least prevalent but often the most severe and refractory form, affecting approximately 2–4% of the general population, [2] and 15–30% of patients referred to tertiary motility clinics. [3] STC is characterized by delayed colonic transit in the absence of mechanical obstruction and reflects abnormalities of colonic motor activity and neuromuscular function [4].

The pathophysiology of STC remains incompletely understood [3, 5]. Several abnormalities have been described, including degeneration of myenteric plexus ganglia and broader neuromuscular dysfunction. [5] Additionally, alterations in the enteric nervous system and a reduction in interstitial cells of Cajal have been reported, in the absence of identifiable secondary causes [6].

While most patients with CC respond to conservative management, including laxatives, [7] advanced pharmacological therapy, [8] and non-drug interventions such as behavioural therapy, biofeedback, or transanal irrigation, [9] a small but clinically relevant subset of patients with STC remains refractory to initial treatments. In these carefully selected cases, colonic resection has been advocated as a viable therapeutic option.

Surgical approaches to severe constipation date back to the early twentieth century, [10] when Sir Arbuthnot Lane advocated colectomy for ‘chronic intestinal stasis’, [11] a controversial theory linking constipation to systemic autointoxication that led to surgical treatment of medical conditions including fatigue or epilepsy [12]. While strongly debated and later criticised, [12, 13] these radical procedures represented some of the earliest attempts to surgically address conditions corresponding to what is now recognised as STC.

Subsequently, surgical strategies evolved and total colectomy with ileorectal anastomosis (TC-IRA) became the standard surgical treatment for carefully selected patients with STC [14]. Nevertheless, outcomes remain heterogeneous and the procedure is associated with significant perioperative morbidity and long-term functional sequelae [15]. These limitations have fuelled the development of alternative options aimed at preserving part of the colon while maintaining adequate bowel function [15]. Among these, Sarli and colleagues described subtotal colectomy with antiperistaltic caecorectal anastomosis (SCCRA) as a technique designed to avoid the extensive right colonic mobilization and counterclockwise rotation of the right colon required in the Deloyers reconstruction, [16] while preserving the ileocecal junction [17]. Preservation of the ileocecal junction may help maintain absorptive capacity and mitigate postoperative diarrhoea, given its physiological role in bile acid, vitamin B12, and electrolyte absorption [18, 19]. The adoption of minimally invasive techniques may further improve perioperative outcomes [20, 21].

However, current evidence on SCCRA remains limited to single reports, retrospective series and small prospective studies, with scarce comparative data against TC-IRA.

The present study aimed to evaluate the clinical and functional outcomes following minimally invasive SCCRA in patients with refractory STC and to compare these outcomes with those of an independent cohort treated with TC-IRA.

## Methods

### Study design

This study was designed as a prospective bicentre cohort study evaluating clinical and functional outcomes following minimally invasive SCCRA in patients with refractory STC. To provide contextual comparison with the current standard surgical treatment, a retrospective cohort of patients treated with TC-IRA was also analysed.

The study was approved by the institutional ethics committee and conducted in accordance with the Declaration of Helsinki. All patients provided written informed consent for the surgical procedure and for the use of anonymized clinical data for research purposes. Patient data were anonymized prior to analysis. STROBE guidelines for observational cohort studies were followed.

### Setting

Patients undergoing SCCRA were consecutively enrolled at two tertiary referral centres.

Patients undergoing TC-IRA were identified from a prospectively maintained institutional database at a third referral centre and served as a historical comparison cohort.

SCCRA procedures were performed between April 2023 and June 2025, while TC-IRA procedures were performed between March 2013 and August 2025.

Follow-up duration was calculated from the date of surgery to the latest available clinical assessment.

### Participants

Consecutive adult patients undergoing surgery for refractory STC were eligible. The diagnosis of STC was established through radiopaque marker colonic transit testing after exclusion of organic colorectal disease and clinically relevant evacuation disorders. Preoperative assessment included colonoscopy to exclude colorectal neoplasia, inflammatory bowel disease, mechanical obstruction, or other organic colonic disease. In addition, all patients underwent barium defecography to assess anorectal evacuation. Clinically significant anatomical abnormalities of the rectum, major functional evacuation disorders, and megarectum were considered contraindications to colectomy. STC was confirmed using the standard single-capsule radiopaque marker test (Sitzmarks^®^, Konsyl Pharmaceuticals, USA). Patients ingested one capsule containing 24 radiopaque markers, followed by a plain abdominal radiograph 120 h later. Delayed colonic transit was defined as retention of ≥ 5 markers, corresponding to > 20% of the ingested markers, in accordance with clinical practice guidelines [22]. Postoperative transit studies were not routinely repeated, as the predefined outcomes of the present study were clinical, functional, and quality-of-life measures. Follow-up assessments were performed during scheduled outpatient visits.

### Inclusion criteria

Patients were included if they were aged 18 years or older, had a diagnosis of STC confirmed by objective colonic transit testing, had persistent symptoms despite optimized conservative treatment including laxatives, other pharmacological and non-pharmacological measures, had undergone comprehensive preoperative assessment, and were considered suitable candidates for colectomy according to contemporary clinical practice recommendations, [15, 23] and previously published selection frameworks for colectomy in STC [24].

### Exclusion criteria

Patients were excluded if they had normal colonic transit or unproven STC, inflammatory bowel disease, colorectal neoplasia, mechanical bowel obstruction, or other organic colonic disease, untreated evacuation disorder or other major anorectal dysfunction considered to contraindicate colectomy, clinically significant faecal incontinence or impaired sphincter function precluding restorative colectomy, major extrinsic or systemic causes of constipation judged to make colectomy inappropriate.

### Variables and data collection

Baseline variables included age at surgery, age at symptom onset, comorbidities, ongoing pharmacological therapy, obstetric history (including parity and mode of delivery), and preoperative laxative use.

Operative variables included operative time, surgical approach, number of trocars, stapler size, and specimen extraction site.

Postoperative variables included length of hospital stay, complications, and need for stoma formation. Postoperative complications were classified according to the Clavien-Dindo classification [25].

In the SCCRA cohort, functional outcomes were prospectively assessed both preoperatively and during postoperative follow-up and included the Patient Assessment of Constipation-Symptoms (PAC-SYM), [26] the Patient Assessment of Constipation-Quality of Life (PAC-QOL), [27] St Mark’s incontinence score, [28] weekly bowel movement frequency, and laxative use. These outcomes were collected using validated patient-reported questionnaires administered preoperatively and during follow-up visits. Postoperative questionnaires were administered by an independent assessor not involved in the surgical care of the patients.

For the historical TC-IRA cohort, only PAC-QOL data were available and were extracted retrospectively from a prospectively maintained institutional database.

## Surgical techniques

### SCCRA cohort

Patients underwent minimally invasive subtotal colectomy (laparoscopic or robotic according to surgeon preference and institutional availability) with preservation of the caecum and ileocecal valve, followed by construction of an antiperistaltic end-to-end caecorectal anastomosis, as previously described [20, 21].

Key operative principles included preservation of the ileocolic vascular pedicle, avoidance of mesenteric torsion, and creation of a stapled antiperistaltic caecorectal anastomosis.

### TC-IRA cohort

Control patients underwent TC-IRA using open or minimally invasive approaches according to standard surgical practice. The procedure consisted of removal of the entire colon followed by ileorectal reconstruction, ensuring a well-perfused and tension-free anastomosis while preserving rectal function.

### Data sources

Clinical, operative, and follow-up data were obtained from prospectively maintained electronic medical records. Patient reported outcome measures were prospectively collected in the SCCRA cohort, whereas PAC-QOL data for the TC-IRA cohort were extracted from a prospectively maintained institutional database.

### Outcomes and functional assessment

The primary outcome of the study was the change in PAC-QOL score between baseline and the 6-month postoperative assessment.

Secondary outcomes included changes in PAC-SYM score, St Mark’s incontinence score, weekly bowel movement frequency, and postoperative laxative use, and histological assessment of surgical specimens.

In the SCCRA cohort, patients completed the PAC-SYM and PAC-QOL questionnaires preoperatively and at 6 months after surgery. In the TC-IRA cohort, PAC-QOL was also recorded at 6 months postoperatively.

Additional outcome measures, including weekly bowel movement frequency and postoperative laxative use, were assessed at the latest available postoperative follow-up.

Comparative analyses between SCCRA and TC-IRA cohorts were performed for baseline characteristics, operative variables, and postoperative outcomes. Functional comparisons between the two cohorts were limited to PAC-QOL scores, which were available in both datasets, and restricted to patients with restored bowel continuity.

### Histopathological analysis

Histopathological reports from colectomy specimens were retrospectively reviewed to characterize neuromuscular abnormalities of the colonic wall. Findings were extracted from routine pathology reports and recorded descriptively.

### Bias

Several measures were undertaken to limit potential sources of bias: prospective enrolment of consecutive patients in the SCCRA cohort, application of objective diagnostic criteria for STC, and use of validated functional outcome instruments. However, comparison with the TC-IRA cohort should be interpreted cautiously given the retrospective design and historical nature of the control cohort, as patient selection may not have fully reflected more recent graded practice recommendations for colectomy in STC.

### Statistical analysis

Continuous variables were summarized as median and interquartile range (IQR), while categorical variables were expressed as absolute numbers and percentages. Given the expected non-normal distribution and limited sample size, non-parametric statistical tests were used. Between-group comparisons were performed using Mann-Whitney U test for continuous variables, χ² test or Fisher’s exact test for categorical variables. Within-group comparisons of preoperative and postoperative functional scores were performed using the Wilcoxon signed-rank test. Analyses were performed on available data. Missing values were not imputed. Only patients with available follow-up data were included in functional outcome analyses. A two-sided p value < 0.05 was considered statistically significant. Statistical analyses were performed using SPSS version 30 (IBM Corp., Armonk, NY, USA).

## Results

### Patient population

**Fig. 1:**
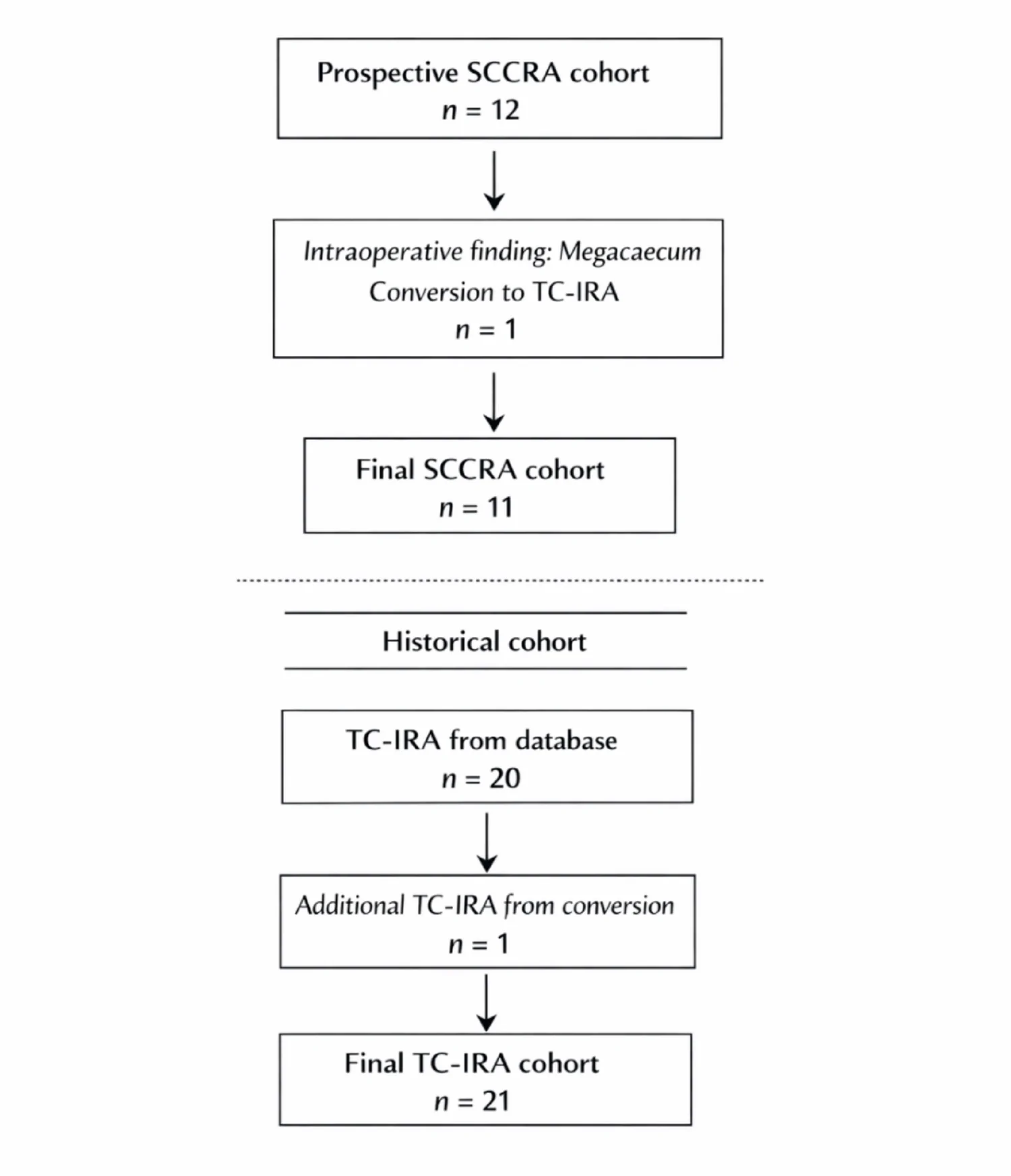
Flow diagram of patients’ inclusion

Twelve consecutive patients were prospectively enrolled for SCCRA. In one patient, intraoperative finding of a markedly dilated caecum precluded safe construction of a caecorectal anastomosis and the procedure was converted to TC-IRA. Therefore, 11 patients underwent SCCRA and were included in the primary cohort (Fig. 1).

### Baseline characteristics of the SCCRA cohort

Of the 11 patients, 9 (82%) were female. Median age at surgery was 51 years (IQR 43–60), and at symptom onset was 30 years (IQR 15–48). Among female patients, two (22%) were nulliparous, while the median parity among parous women was 1 (IQR 1–1). Among parous women, 6/7 had a history of vaginal delivery, whereas one had a previous caesarean section.

Five patients (45%) had comorbidities requiring ongoing pharmacological treatment, including minor depressive disorder (*n* = 3), fibromyalgia (*n* = 1), and hypertension (*n* = 1).

All patients were receiving regular laxative therapy prior to surgery. At baseline, patients reported a median of 1 bowel movement per week (IQR 1–2). Median St Mark’s incontinence score was 0 (IQR 0–0). Median PAC-SYM and PAC-QOL scores were 3.0 (IQR 2.83–3.33) and 2.0 (IQR 1.13–2.43), respectively.

### Operative and postoperative outcomes of SCCRA

Ten procedures were performed laparoscopically and one using a robotic approach with the Versius^®^ surgical system (CMR Surgical, Cambridge, UK) [21]. Among the laparoscopic cases, nine were performed using a conventional multiport technique and one using a single-incision approach [20]. The median number of trocars used in multiport procedures was 5 (IQR 5–6).

Median operative time was 257 min (IQR 190–281). Specimen extraction was performed through a transumbilical (*n* = 5) or Pfannenstiel incision (*n* = 6). Circular stapler size for the caecorectal anastomosis varied between 28 mm (*n* = 2), 29 mm (*n* = 3), 31 mm (*n* = 3), and 32 mm (*n* = 3).

Median length of hospital stay was 4 days (IQR 3.5–6). One patient developed an anastomotic leak requiring reoperation five days after the index procedure. The anastomosis was dismantled with resection of the ileocolic segment and creation of a terminal ileostomy, performed laparoscopically. Eight months later, the patient underwent laparoscopic reconstruction with IRA and a diverting loop ileostomy, followed by ileostomy closure three months thereafter.

A late postoperative event was observed in another patient, who became pregnant 2 years after SCCRA. Approximately 3 months postpartum, she developed bowel obstruction secondary to volvulus of the caecorectal anastomosis, which was successfully treated endoscopically with decompression. She remains recurrence-free 8 months after the event.

### Functional outcomes after SCCRA

Median follow-up was 18 months (IQR 10.0–26.5), ranging from 6 to 34 months.

The PAC-SYM and PAC-QOL scores significantly improved following surgery, decreasing to 0.67 (IQR 0.67–1.00, *p* = 0.004) and 0.07 (IQR 0.00–0.36, *p* < 0.001) at 6-month follow-up, respectively.

The St Mark’s incontinence score remained 0 (IQR 0–0) at last follow-up in all patients. The number of bowel movements per week significantly increased from a median of 1 (IQR 1–2) preoperatively to 7 (IQR 5–7) at last follow-up (*p* = 0.003).

### Characteristics of the TC-IRA cohort

The TC-IRA cohort included 21 patients, of whom 17 (81%) were female. Median age at surgery was 59 years (IQR 52–64), and at symptom onset was 30 years (IQR 29.5–35). Several patients had relevant comorbidities requiring pharmacological treatment, including minor depression (*n* = 2), phantom limb chronic pain (*n* = 1), multiple sclerosis (*n* = 1), bipolar disorder (*n* = 1), intellectual and motor disability (*n* = 1), autoimmune disease (*n* = 1), and a history of eating disorder (*n* = 1). All patients were receiving regular laxative therapy prior to surgery.

Among female patients, 7 (33%) were nulliparous. Among parous women, the median parity was 1 (IQR 0–1), with one patient having a previous caesarean section. Median baseline PAC-QOL score was 3.88 (IQR 3.57–4.00), and patients reported a median of 1 bowel movement per week (IQR 1–1) preoperatively.

Median operative time was 255 min (IQR 250–270). Seven procedures were performed laparoscopically using 5 trocars, whereas 14 were performed using an open approach. In laparoscopic cases, specimen extraction was performed through a Pfannenstiel (*n* = 6) or a transumbilical incision (*n* = 1).

Median follow-up was 27 months (IQR 15–51), ranging from 7 to 123 months. Postoperative complications occurred in 4 (19%) patients, three of whom required reoperation (two anastomotic leaks and one splenectomy due to splenic laceration). One additional patient developed an intra-abdominal collection treated with computed tomography-guided drainage. Of the patients with a diverting stoma after anastomotic leak, one underwent stoma reversal after 4 months, while the other elected to maintain a permanent ileostomy, and was therefore not included in functional outcome analyses.

### Comparative analysis between SCCRA and TC-IRA

There were no statistically significant differences in median age at surgery (*p* = 0.126) or age at symptom onset (*p* = 0.933) between the two groups. Operative time was also comparable (*p* = 0.735).

Baseline disease burden, as assessed by PAC-QOL, was significantly higher in the TC-IRA cohort (*n* = 20 for functional analyses) compared to the SCCRA group (3.88 [IQR 3.57–4.00] vs. 2.00 [IQR 1.13–2.43], *p* < 0.001). The absolute improvement in PAC-QOL (ΔPAC-QOL) was significantly greater in the TC-IRA group (median 3.28 [IQR 2.25–3.66]) compared to the SCCRA cohort (median 1.54 [IQR 1.10–2.32], *p* < 0.001).

From a functional perspective, among patients with restored bowel continuity, SCCRA was associated with a significantly lower number of bowel movements per week at follow-up compared with TC-IRA (median 7 [IQR 6–7] vs. 21 [IQR 14–28], *p* = 0.002).

### Histopathological analysis

**Fig. 2:**
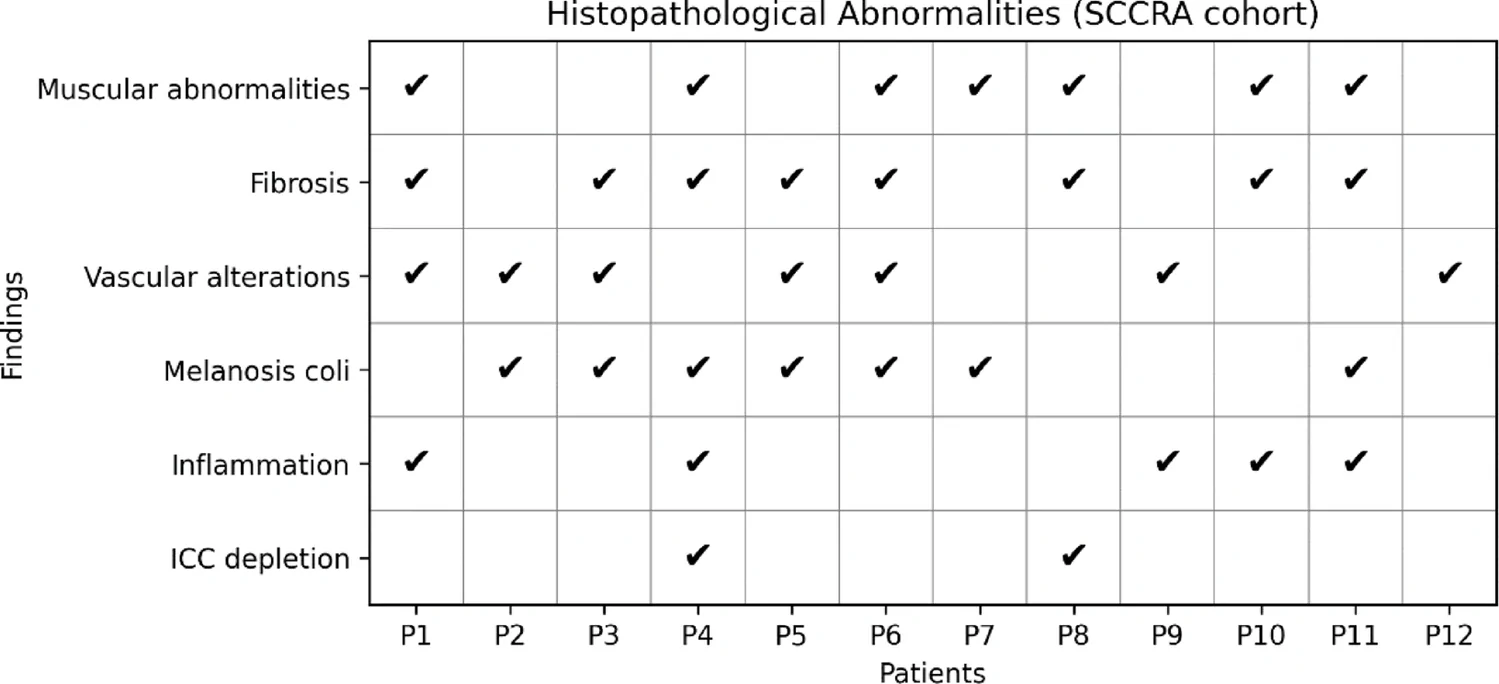
Distribution of histopathological abnormalities across individual patients in the SCCRA cohort

Histopathological examination of colectomy specimens from all patients initially undergoing SCCRA (*n* = 12), including the case converted intraoperatively to TC-IRA, revealed heterogeneous and overlapping structural abnormalities involving different layers of the colonic wall (Fig. 2).

The median specimen length was 76 cm (IQR 68.5–83.5). Muscular layer abnormalities were observed in 7 patients (58%), including atrophy, hypertrophy, disarray, or thinning of the muscularis propria. Fibrotic changes involving either the submucosa or muscular layer were also identified in 7 patients (58%). Vascular alterations, including angiectasia and congestion, were present in 7 patients (58%).

Melanosis coli was a frequent finding, observed in 7 patients (58%). Inflammatory changes were identified in 5 patients (42%), ranging from mild lamina propria inflammation to more specific entities such as lymphocytic myenteric ganglionitis.

Abnormalities involving the enteric nervous system were limited, with only one case showing features of ganglionitis, while most specimens demonstrated preserved ganglion cell architecture.

Immunohistochemical evidence of reduced interstitial cells of Cajal was observed in 2 patients (17%).

## Discussion

SCCRA was associated with significant improvement in constipation-related symptoms and quality of life, as demonstrated by reductions in PAC-SYM and PAC-QOL scores. Bowel function improved substantially after SCCRA, with a median of 7 bowel movements per week, while continence was preserved in all patients. When compared with TC-IRA, SCCRA resulted in a lower postoperative bowel frequency, suggesting a more physiological functional outcome. Finally, histopathological analysis revealed heterogeneous structural abnormalities of the colonic wall, without a single dominant pathological pattern, consistent with previous literature [3, 5].

The observed improvement in patient-reported outcomes following SCCRA is consistent with the rationale of removing the functionally impaired colon while preserving the ileocaecal junction and the caecum. Preservation of the ileocaecal junction may help prevent retrograde bacterial reflux into the terminal ileum, whereas preservation of the caecum may contribute to continued water and electrolyte absorption and provide a reservoir function. Together, these mechanisms may contribute to the more physiological bowel frequency observed in this cohort [29, 30]. Compared to TC-IRA, SCCRA appears to provide a balance between symptom relief and preservation of bowel function, potentially reducing the risk of postoperative diarrhoea and urgency.

In the comparative analysis, the greater absolute improvement in PAC-QOL observed in the TC-IRA group should be interpreted in light of the significantly higher baseline disease burden. This suggests a potential ceiling effect, whereby patients with more severe preoperative impairment have greater room for improvement. Therefore, differences in ΔPAC-QOL between groups may reflect baseline imbalance rather than a true superiority of one procedure over the other.

The histopathological findings further support the concept of STC as a heterogeneous and multifactorial disorder. Abnormalities involving the muscular layer, fibrosis, vascular changes, and, less frequently, enteric nervous system alterations were variably present across specimens. The absence of a consistent dominant pattern suggests that STC may represent a spectrum of neuromuscular dysfunction rather than a single disease entity, which may partly explain the variability in surgical outcomes reported in the literature.

This study has several strengths. The SCCRA cohort was prospectively enrolled, with consecutive inclusion of patients and use of validated patient-reported outcome measures collected at predefined time points. In addition, postoperative functional assessment was performed by an independent assessor, reducing the risk of observer bias.

However, several limitations should be acknowledged. First, the comparison with the TC-IRA cohort is limited by its retrospective design and historical nature, which may introduce selection bias and confounding due to differences in patient characteristics, surgical indications, and perioperative management over time. In particular, the significantly higher baseline PAC-QOL scores in the TC-IRA group indicate a difference in disease severity that may have influenced comparative results.

Second, the sample size of the SCCRA cohort is relatively small, reflecting the rarity of surgically treated STC and the strict selection criteria applied [24]. This limits the statistical power of the study and the ability to perform adjusted analyses. Third, functional outcomes in the two cohorts were not fully comparable, as only PAC-QOL data were available for both groups, precluding a comprehensive comparison of all functional domains.

Finally, histopathological analysis was descriptive and based on routine pathology reports rather than standardized or blinded reassessment, which may limit the consistency and reproducibility of these findings.

The findings of this study suggest that SCCRA may represent a viable alternative to TC-IRA in selected patients with refractory STC, particularly when preservation of a more physiological bowel function is a priority. However, careful patient selection remains crucial, given the heterogeneity of the underlying pathophysiology.

Future research should focus on high-quality prospective comparative studies with standardized functional assessments. Notably, a large multicentre randomized controlled trial comparing TC-IRA and SCCRA (STOPS trial) is currently ongoing and may provide more robust evidence to guide surgical decision-making in this setting [31].

## Conclusions

Minimally invasive SCCRA provides significant improvement in symptoms and quality of life in patients with refractory STC, while maintaining a more physiological bowel frequency compared with TC-IRA. The heterogeneous histopathological findings observed in this study reinforce the concept of STC as a multifactorial disorder. These results should be interpreted cautiously due to baseline differences and the non-randomized study design.

## Data Availability

The datasets generated and analysed during the current study are available from the corresponding authors on reasonable request.

## References

[1] Corsetti M, Shin A, Lacy BE, Cash BD, Simrén M, Schmulson MJ, Hou X, Lembo A (2026) Bowel disorders. Gastroenterology 170(6):1261–1282. 10.1053/j.gastro.2026.02.003 41713703

[2] Bharucha AE, Lacy BE (2020) Mechanisms, evaluation, and management of chronic constipation. Gastroenterology 158:1232–1249 e3 10.1053/j.gastro.2019.12.034 PMC7573977 31945360

[3] Syllaios A, Papadakos SP, Ioannou A, Frountzas M, Michelakis D, Patsouras D, Dritsas S, Pramateftakis MG, Schizas D (2026) Slow transit constipation: Pathophysiological perspectives and management updates. J Dig Dis 27(1–2):15–30. 10.1111/1751-2980.70030 PMC13097960 41646037

[4] Scott SM, Simren M, Farmer AD et al (2021) Chronic constipation in adults: contemporary perspectives and clinical challenges. 1: Epidemiology, diagnosis, clinical associations, pathophysiology and investigation. Neurogastroenterol Motil 33:e14050 10.1111/nmo.14050 33263938

[5] Knowles CH, Scott SM, Lunniss PJ (2001) Slow transit constipation: a disorder of pelvic autonomic nerves? Dig Dis Sci 46:389–401 10.1023/a:1005665218647 11281190

[6] He CL, Burgart L, Wang L et al (2000) Decreased interstitial cell of cajal volume in patients with slow-transit constipation. Gastroenterology 118:14–21 10.1016/s0016-5085(00)70409-4 10611149

[7] Colbran R, Neshatian L (2026) Laxative logic: when lifestyle is not enough. Curr Opin Gastroenterol 42:33–42 10.1097/MOG.0000000000001137 41017482

[8] Bor S, Kalkan IH, Savarino E et al (2024) Prokinetics-safety and efficacy: the European society of neurogastroenterology and motility/the American neurogastroenterology and motility society expert review. Neurogastroenterol Motil 36:e14774 10.1111/nmo.14774 38462678

[9] Knowles CH, Booth L, Brown SR et al (2021) Non-drug therapies for the management of chronic constipation in adults: the CapaCiTY research programme including three RCTs. Programme Grants Appl Res 9(14):1–134. 34855315

[10] Lane WA, (1908) Remarks, on the results of the operative treatment of chronic constipation. Br Med J 1:126–130 10.1136/bmj.1.2455.126 PMC2435825 20763645

[11] Lane WA (1912) Chronic intestinal stasis. Lancet 180:1706–1708

[12] Smith JL (1982) Sir Arbuthnot Lane, chronic intestinal stasis, and autointoxication. Ann Intern Med 96:365–369 10.7326/0003-4819-96-3-365 7036818

[13] Hurst AF, (1922) An address, on the sins and sorrows of the colon: delivered before the harrogate medical society on november 26th, 1921. Br Med J 1:941–943 10.1136/bmj.1.3207.941 PMC2416244 20770762

[14] Knowles CH, Scott M, Lunniss PJ (1999) Outcome of colectomy for slow transit constipation. Ann Surg 230:627–638 10.1097/00000658-199911000-00004 PMC1420916 10561086

[15] Knowles CH, Grossi U, Chapman M et al (2017) Surgery for constipation: systematic review and practice recommendations: results I: colonic resection. Colorectal Dis 19(Suppl 3):17–36 10.1111/codi.13779 28960923

[16] Costalat G, Garrigues JM, Didelot JM et al (1997) Subtotal colectomy with ceco-rectal anastomosis (Deloyers) for severe idiopathic constipation: an alternative to total colectomy reducing risks of digestive sequelae. Ann Chir 51:248–255 9297887

[17] Sarli L, Costi R, Sarli D et al (2001) Pilot study of subtotal colectomy with antiperistaltic cecoproctostomy for the treatment of chronic slow-transit constipation. Dis Colon Rectum 44:1514–1520 10.1007/BF02234608 11598483

[18] Levitan R, Fordtran JS, Burrows BA et al (1962) Water and salt absorption in the human colon. J Clin Invest 41:1754–1759 10.1172/JCI104634 PMC291098 14464699

[19] Sarli L, Iusco D, Violi V et al (2002) Subtotal colectomy with antiperistaltic cecorectal anastomosis. Tech Coloproctol 6:23–26 10.1007/s101510200004 12077637

[20] Grossi U, Piccino M, Sarli L (2024) Single-incision laparoscopic Sarli procedure: subtotal colectomy with antiperistaltic caecorectal anastomosis for slow-transit constipation—a video vignette. Colorectal Dis 26:1091–1093 10.1111/codi.16963 38553808

[21] Pecorino A, Grego A, Grossi U et al (2025) Innovative application of the Versius Surgical System(R) in subtotal colectomy for refractory slow-transit constipation: a video vignette. Colorectal Dis 27:e70014 10.1111/codi.70014 39871431

[22] Rao SS, Camilleri M, Hasler WL et al (2011) Evaluation of gastrointestinal transit in clinical practice: position paper of the American and European Neurogastroenterology and Motility societies. Neurogastroenterol Motil 23:8–23 10.1111/j.1365-2982.2010.01612.x 21138500

[23] Knowles CH, Grossi U, Horrocks EJ et al (2017) Surgery for constipation: systematic review and practice recommendations: graded practice and future research recommendations. Colorectal Dis 19(Suppl 3):101–113 10.1111/codi.13775 28960922

[24] Chaichanavichkij P, Vollebregt PF, Tee SZY et al (2021) Slow-transit constipation and criteria for colectomy: a cross-sectional study of 1568 patients. BJS Open 5(3):zrab049. 10.1093/bjsopen/zrab049 PMC8164778 34052848

[25] Dindo D, Demartines N, Clavien PA (2004) Classification of surgical complications: a new proposal with evaluation in a cohort of 6336 patients and results of a survey. Ann Surg 240:205–213 10.1097/01.sla.0000133083.54934.ae PMC1360123 15273542

[26] Frank L, Kleinman L, Farup C et al (1999) Psychometric validation of a constipation symptom assessment questionnaire. Scand J Gastroenterol 34:870–877 10.1080/003655299750025327 10522604

[27] Marquis P, De La Loge C, Dubois D et al (2005) Development and validation of the patient assessment of constipation quality of life questionnaire. Scand J Gastroenterol 40:540–551 10.1080/00365520510012208 16036506

[28] Vaizey CJ, Carapeti E, Cahill JA et al (1999) Prospective comparison of faecal incontinence grading systems. Gut 44:77–80 10.1136/gut.44.1.77 PMC1760067 9862829

[29] Germani P, Zucca A, Giudici F et al (2021) Ileocecal valve syndrome and vitamin b12 deficiency after surgery: a multicentric prospective study. Updates Surg 73:569–580 10.1007/s13304-020-00845-z PMC8005395 32648110

[30] Shafik AA, Ahmed IA, Shafik A et al (2011) Ileocecal junction: anatomic, histologic, radiologic and endoscopic studies with special reference to its antireflux mechanism. Surg Radiol Anat 33:249–256 10.1007/s00276-010-0762-x 21184079

[31] Tian Y, Guo M, Bu F et al (2025) Total colectomy with ileorectal anastomosis versus subtotal colectomy with cecal-rectal anastomosis for slow transit constipation: protocol for a multicenter randomized controlled trial (STOPS trial). Trials 26:402 10.1186/s13063-025-09049-5 PMC12512699 41074220

